# Improving carboxymethyl cellulose edible coating using ZnO nanoparticles from irradiated *Alternaria* *tenuissima*

**DOI:** 10.1186/s13568-022-01459-x

**Published:** 2022-09-07

**Authors:** Mervat M. Anwar, Sanaa S. H. Aly, Essam H. Nasr, El-Sayed R. El-Sayed

**Affiliations:** 1 Plant Research Department, Nuclear Research Center, Egyptian Atomic Energy Authority, Cairo, Egypt; 2 Food Engineering and Packing Department, Agriculture Research Centre, Food Technology Research Institute, Giza, Egypt

**Keywords:** Zinc oxide nanoparticles, *Alternariatenuissima*, Edible coating films, Active food packaging, Carboxymethyl cellulose, Gamma irradiation

## Abstract

In this paper, gamma-irradiation was successfully used to intensify the yield of Zinc oxide nanoparticles (ZnONPs) produced by the fungus *Alternaria* *tenuissima* as a sustainable and green process. The obtained data showed that 500 Gy of gamma-irradiation increased ZnONPs’ yield to approximately four-fold. The synthesized ZnONPs were then exploited to develop active Carboxymethyl Cellulose films by casting method at two different concentration of ZnONPs 0.5% and 1.0%. The physicochemical, mechanical, antioxidant, and antimicrobial properties of the prepared films were evaluated. The incorporation of ZnONPs in the Carboxymethyl Cellulose films had significantly decreased solubility (from 78.31% to 66.04% and 59.72%), water vapor permeability (from 0.475 g m^−^^2^ to 0.093 g m^−^^2^ and 0.026 g m^−^^2^), and oxygen transfer rate (from 24.7 × 10^–^^2^ to 2.3 × 10^–^^2^ and 1.8 × 10^–^^2^) of the respective prepared films. Meanwhile, tensile strength (from 183.2 MPa to 203.34 MPa and 235.94 MPa), elongation (from 13.0% to 62.5% and 83.7%), and Yang's modulus (from 325.344 to 1410.0 and 1814.96 MPa) of these films were increased. Moreover, the antioxidant and antimicrobial activities against several human and plant pathogens the prepared of Carboxymethyl Cellulose-ZnONPs films were significantly increased. In conclusion, the prepared Carboxymethyl Cellulose-ZnONPs films showed enhanced activities in comparison with Carboxymethyl Cellulose film without NPs. With these advantages, the fabricated Carboxymethyl Cellulose-ZnONPs films in this study could be effectively utilized as protective edible coating films of food products.

## Introduction

Food products undergo deterioration during production, processing, distribution, and storage. Active packaging, a subset of intelligent packaging, could be used as a solution where many benefits such as maintaining quality, safety, and preservation are provided (Sahraeea et al. 2019). It is simply defined as the inclusion of some special addition(s) either in in‐pack films or packaging (La et al. 2021). Using this approach, several advantages could be obtained including controlling both oxidation and respiration rates, improved moisture migration, odor absorbers, controlling microbial growth, lower carbon dioxide emitting and absorption, and ethylene substances elimination (Ebrahimi et al. 2019). For example, a biopolymer such as carboxymethyl cellulose (CMC) is used in combination with some active substances as a substitute of synthetic plastics to reduce their environmental pollution, however, the hydrophilic properties of these proteins and polysaccharides limited their applications (Han and Wang 2017). So, developments have been made with the introduction of nanotechnology in the packaging industry. In particular, the use of nano-systems (Peighambardoust and Pourabbas 2007; Khodaeimehr et al. 2018) in edible coatings has emerged as promising and successful strategy for food preservation (Zambrano-Zaragoza et al. 2018, and references therein). Films made of nanoparticles (NPs) and biopolymers showed improved properties including mechanical (Oun and Rhim 2017; Dehghani et al. 2019; Peighambardoust et al. 2019) and antimicrobial properties (Fasihnia et al. 2018; Hajizadeh et al. 2020). Thus, the interest in using biocompatible NPs in the field of active food packaging will continue to rise.

Zinc is one of the most important essential elements for animals and humans. It plays a central role in maintaining cell cycle progression and several cellular processes such as oxidative stress as well as DNA repair and replication (Bisht and Rayamajhi 2016). Recent studies proved the superiority of Zinc oxide NPs (ZnONPs) compared to the conventional sources of Zinc. It exhibited considerably low toxicity (Abdelhakim et al. 2020), high absorption rate (Yusof et al. 2019), improved bioavailability (Hosseini and Sarviab 2015), and better biocompatibility (Shahid et al. 2019). As such, a wide range of therapeutic and biological activities such as anticancer, antibacterial (Abdelhakim et al. 2020), antifungal, antioxidant (Mousa et al. 2021), anti-protozoa (Wajiha et al. 2018). Thus, these NPs could be a promising candidate in the field of active packaging owing to these unique properties.

Traditionally, various physical as well as chemical technologies are used to prepare ZnONPs. In spite of that such technologies are costly and complicated besides the generation of hazardous side-streams with negative impacts not only on the health but also the environment. In this regard the microbial synthesis of ZnONPs has magnificently emerged as a promising alternative, nevertheless, it remains unexplored till now. In the literature, few studies have reported the synthesis of these NPs by some microbial strains (Mousa et al. 2021) . A recent study proved the ability of the endophytic fungus *Alternaria* *tenuissima* to prepare ZnONPs with promising antioxidant and antimicrobial activities (Abdelhakim et al. 2020). Essentially, scaling up the synthesis of nanomaterials using microorganisms and the subsequent development of a suitable applicative process are emerging and attractive prospects for a sustainable production in the near future. Thus, we aim in this paper to intensify the potentiality of this promising fungus using gamma-irradiation as a bio-factory for ZnONPs. In the same connection, the toxic chemicals required in the preparation of ZnONPs by chemical approaches greatly reduce their applications (Yusof et al. 2019). Therefore, the bio-inspired ZnONPs in this study were exploited in the development of active CMC-based coating films for the first time. All the physicochemical, mechanical, antioxidant, antibacterial, and antifungal properties of the prepared CMC-based ZnONPs films were studied.

## Materials and methods

### Synthesis and characterization of ZnONPs

Synthesis of ZnONPs was performed using the fungus *Alternaria* *tenuissima* AUMC10624 (Culture Collection of Assiut University Mycological Center, Assiut, Egypt, http://www.aun.edu.eg/aumc/aumc.htm) according to the method described in details in previous study (Abdelhakim et al. 2020). In brief, cell-free culture filtrate of the fungus *A.* *tenuissima* was mixed with aqueous solution of 2 mM zinc sulfate (Sigma-Aldrich, USA) in equal volume basis. Then, the mixture kept at room temperature under vigorous stirring for 20 min. The resultant white precipitate (ZnONPs) was separated from the mixture by ultracentrifugation, washed in deionized water, ethanol and dried at 50 °C. The fine powders of ZnONPs were then dissolved in ethanol (HPLC grade), treated ultrasonically (for the dispersion of the individual NPs), then characterized. Characterization was accomplished by the following techniques: X-ray diffraction (XRD, D8 DISCOVER with DAVINCI design, USA), Zeta potential and Dynamic light scattering (DLS) analyses (Zetasizer Nano ZS, Malvern Instruments, Worcestershire, UK), and Transmission Electron Microscope operated at an accelerating voltage at 8000 kV (JOEL model 2100, Japan).

### Influence of Co^60^ gamma irradiation on the production of ZnONPs

The effect of different gamma-irradiation doses from 250 to 4000 Gy on the preparation of ZnONPs by the culture-free filtrate of the fungus was studied. Irradiation process was performed by exposing spore suspensions of the fungus to gamma rays in a ^60^Co Gamma chamber (MC20, Russia, the average dose rate was 605.726 Gy h^−^^1^) at the Nuclear Research Center, Egyptian Atomic Energy Authority. The irradiated spore suspensions were kept in darkness overnight (at 4 °C) to prevent the photo-reactivation. Then, 1 mL of each radiation dose was inoculated to potato-broth medium and incubated for 10 days at 30 °C. The cell-free filtrates resulted from each irradiation dose were separately used to prepare ZnONPs, as described earlier. The yield of ZnONPs of each irradiation dose was calculated after recording the UV-absorption at 369 nm (UV–Vis spectrophotometer, UV-3101PC, Shimadzu, Japan) and expressed as OD mL^−^^1^. Moreover, the resultant powders from every dose were carefully weighed and the yield was expressed as mg ZnONPs 100 mL^−^^1^.

### Preparation of CMC-ZnONPs films

Carboxymethyl cellulose (CMC, purchased from TechnoGene Company, Giza, Egypt) aqueous solution was prepared by dissolving 2% (w/v) of CMC powder in distilled water at 75 °C under the high-speed mixer (900 rpm) for 15 min. Then, glycerol (1.5%, W/V) was added and the solution was stirred for another 10 min under the same conditions. Then, two concentrations of CMC-ZnONPs, 0.5 and 1.0% (w/v) were prepared separately by adding 5 and 10 g ZnONPs powder to the prepared CMC solution and stirred for 15 min. The two concentrations were selected based on a preliminary experiment concerning the homogeneity and ease of casting. The prepared mixtures were kept in an ultrasonic bath for 30 min, to ensure their homogeneity. After which, the mixtures were poured on the Plexiglas then the wet films were dried at 25 ℃ and 50% relative humidity in a specific oven (Memmert, Germany) for 24 h. Finally, the dried films were separated from Plexiglas then kept in a desiccator (50–60% relative humidity) until the films were tested.

### Determination of physicochemical properties of the prepared films

The thickness of the films was measured using a digital micrometer (Model pk-1012 E, Mitutoyodigimatic indicator corporation, Japan). The film strips were set down between jaws of the micrometer then the gaps were decreased slowly till the first contact was observed (Tien et al. 2000).

Water solubility of the prepared films was calculated as follows: the films were first cut into square pieces (1 cm × 1 cm) then dried in a vacuum oven (at 60 °C) to a constant weight for 24 h to record the initial dry weight of the film (W_*d*_). After which, the film was then introduced into a bottle containing 20 mL distilled water and kept under gentle agitation for at 25 °C for 24 h. The film was finally dried under the before-mentioned conditions to record the dry weight of the water-leached film (W_*ws*_). Water solubility of the film was calculated using the following formula:

$${\text{Water solubility }}\left( \% \right) \, = \, \left( {{\text{W}}_{d} - {\text{ W}}_{ws} } \right)/{\text{W}}_{{\text{d}}} \times { 1}00.$$

The water vapor transmission rate (WVTR) and water vapor permeability (WVP) of the films were measured gravimetrically according to the ASTM method E96-95 (ASTM 1990). In which, the film was first to cut into a circular shape using a circular test cup that was larger than the inner diameter of the cup. Then, the cup was filled with distilled water, sealed at the top using paraffin oil, and placed in a desiccator. The weight of the cup was recorded regularly (every hour) for 10 h and the film specimens were tested. A Linear regression was used to determine slope of this line in g/h. Values of WVTR and WVP were calculated using the following formulas:

$${\text{WVTR}} = \, \Delta {\text{m}}/\Delta {\text{tA,}}$$

$${\text{WVP}} = {\text{WVPR}}.{\text{L}}/\Delta R{\text{H,}}$$

where, ∆*m*/∆*t* represents the moisture gain weight per time (g/s), A represents the film surface area, L represents the film thickness in mm, and ∆*R*H represents difference in the relative humidity.

The transparency of the film samples was measured by a Light transmittance meter–Linshang. The oxygen transmission rate was measured using Gas Permeability Analyzer for Testing Packaging Material, GTR N530-B, GBPI, Guangzhou city, China.

### Determination of mechanical properties

Mechanical properties of Young's modulus, tensile strength, and elongation at break for the prepared films were measured by a Brookfield CT3 Texture Analyzer, Brookfield, USA*.* The films were cut into strips of 3 × 5 cm and gripped at each end by a jaw, and then the jaws were moved at the controlled speed until the modulus was automatically recorded (Hernandez-Mun et al. 2004).

### Antioxidant activity of the prepared films

Free radical scavenging potentials of the CMC, CMC + 0.5%ZnONPs, and CMC + 1.0%ZnONPs films were individually estimated by 2,2^\^-diphenylpicrylhydrazyl (DPPH, Sigma-Aldrich, USA) free radical scavenging assay (El-Sayed et al. 2020a). Film samples were dissolves in distilled water, added separately to the DPPH solution and the percentage of scavenging activities were expressed as the change in the recorded absorbance of the mixture (DPPH + film solution) versus the control (containing DPPH only). Under the same conditions, a positive control of ascorbic acid (Sigma-Aldrich, USA) was tested.

### Antimicrobial sensitivity tests of the prepared films

Antimicrobial potentials of the CMC, CMC + 0.5%ZnONPs, and CMC + 1.0%ZnONPs films were studied using the agar well diffusion assay (El-Sayed et al. 2020b) against different Gram-negative and Gram-positive bacterial strains (*Escherichia* *coli* ATCC11229 and *Staphylococcus* *aureus* ATCC6538), *Candida* *albicans* ATCC10231, *Aspergillus* *brasiliensis* ATCC16404, and a plant pathogenic fungus *Fusarium* *oxysporum* EUM37. Control Petri-dishes were made by applying azithromycin (antibiotic) or ketoconazole (antifungal) to the agar wells. Inhibition zones around the agar wells were carefully measured.

### Statistics

Experimental results were gives as a mean from three independent experiments ± standard deviation. ONE-WAY ANOVA test followed by Least Significant Difference test (at 0.05 level) were used to evaluate the significance using the software SPSS, v 22, IBM Corp.

## Results

### Synthesis and characterization of ZnONPs

**Fig. 1:**
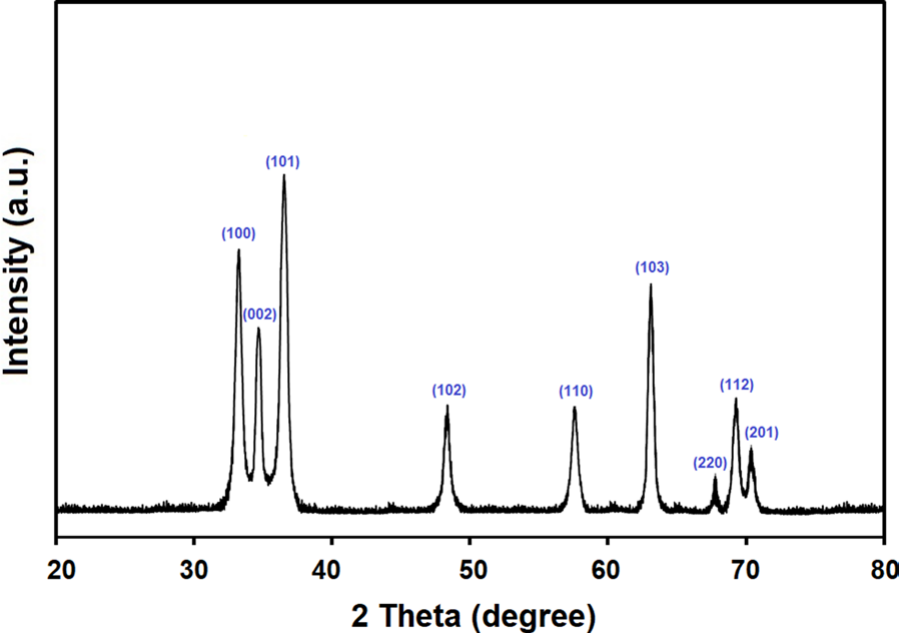
X-ray diffraction pattern (Cu Kα-radiation) at room temperature of ZnONPs synthesized by the fungus *A.* *tenuissima*

**Fig. 2:**
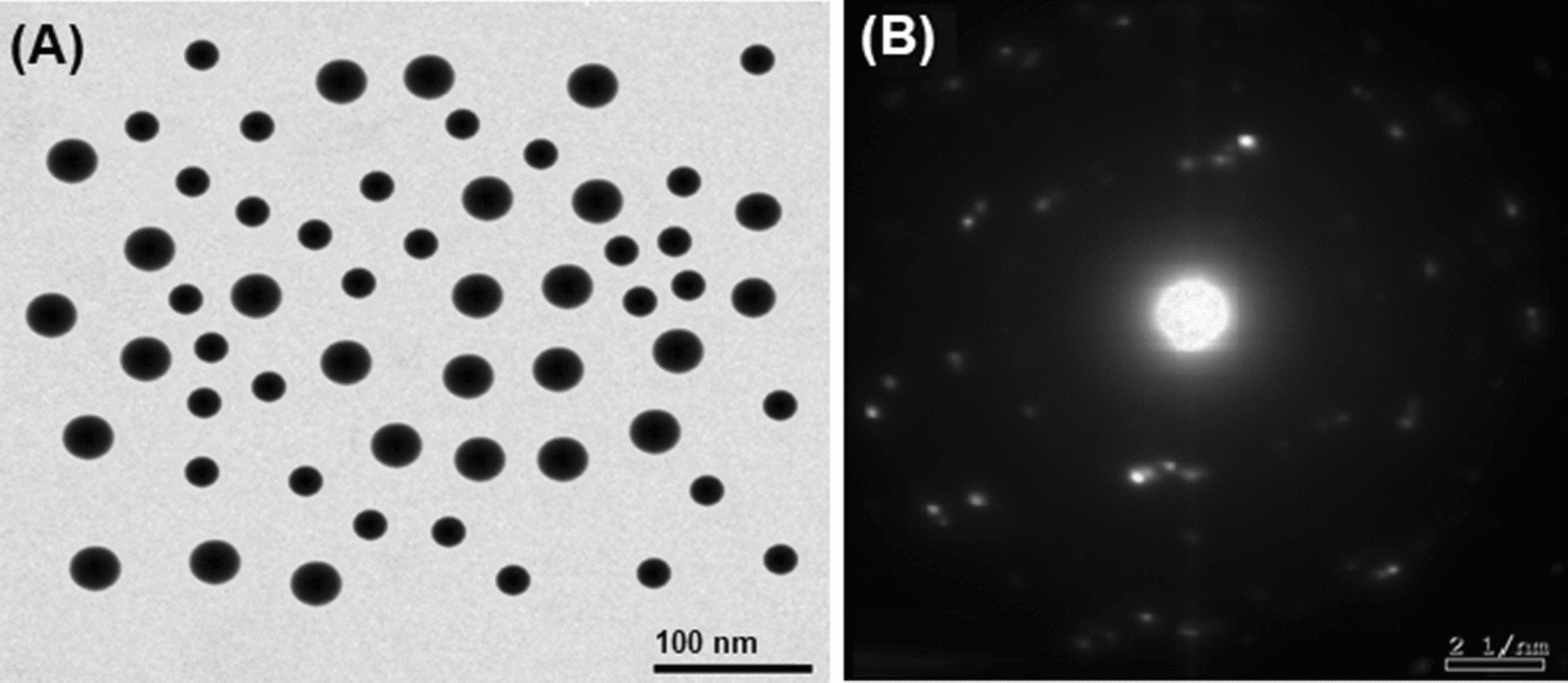
TEM analysis of ZnONPs synthesized by the fungus *A.* *tenuissima*; (**A**) TEM micrograph and (**B**) SAED pattern

**Fig. 3:**
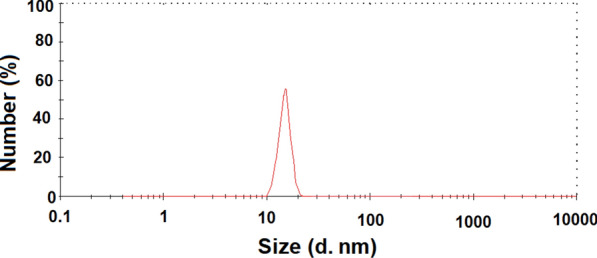
Particle size distribution of ZnONPs synthesized by the fungus *A.* *tenuissima*

**Fig. 4:**
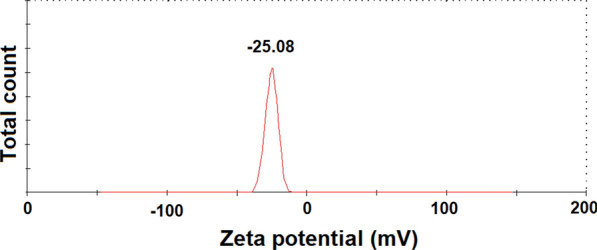
Zeta potential of ZnONPs synthesized by the fungus *A.* *tenuissima*

Figure 1 presents the obtained pattern from the XRD analysis of the prepared ZnONPs. The obtained results confirmed the hexagonal type crystal structure of the prepared ZnONPs with space group P36mc. The planes 100, 002, 101, 102, 110, 103, 220, 112, and 201 in the recorded pattern confirmed the ZnONPs structure. Moreover, the synthesized NPs are pure and of a single phase where no peaks were found in the obtained pattern corresponding to impurities (Fig. 1). Scherrer equation were used to calculate the crystallite size of ZnONPs (from the FWHM of the most intense peak) where it was 15.61 nm. Figure 2 presents the particle size distribution as well as the morphology of the prepared ZnONPs obtained from TEM analysis. TEM images (Fig. 2a) indicated that all the prepared particles were typically spherical in shape. In addition, the SAED pattern presented in Fig. 2b confirms the five rings of the lattice planes corresponding to 100, 002, 101, 110, and 103. Figure 3 presents the particle size distribution in nm of the prepared ZnONPs from the dynamic light scattering analysis. It was noted from the obtained data that (Fig. 3) distribution of the prepared ZnONPs was in the range from 10 to 23 nm. Furthermore, the recorded polydispersity index value was 0.337 which confirm the mono-dispersion of the prepared ZnONPs. Figure 4 shows the zeta potential value of the prepared ZnONPs at -25.08 mV, indicating their stability.

### Production enhancement of ZnONPs by gamma-irradiation of the fungus

**Fig. 5:**
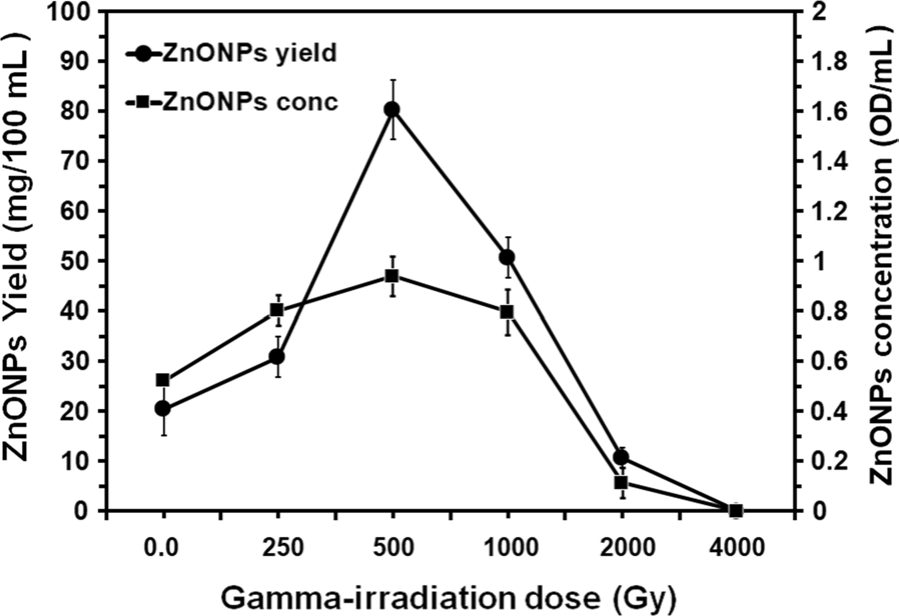
Effect of several gamma-irradiation doses on the production of ZnONPs synthesized by the fungus *A.* *tenuissima*. All data are shown as the mean ± SD of triplicate measurements from three independent experiments

Data presented in Fig. 5 show the influence of exposure of spores of the fungus to gamma rays at several doses on the obtained yield of ZnONPs. The recorded results demonstrated that the dose-related effect of this exposure to gamma rays where a gradual increase in the recorded yield of ZnONPs till a maximum value, then a decline was observed. A such, spore suspension irradiated at 500 Gy intensified the yield of ZnONPs where significant differences where obtained at this dose. The maximum yield expressed by the 0.952 OD mL^−^^1^ culture filtrate and the 81.47 mg/100 mL^−^^1^ culture filtrate of the prepared ZnONPs was reached at this dose; representing approximately a fourfold increase. Meanwhile, increasing irradiation dose to 2000 and 4000 Gy significantly reduced the yield of the prepared ZnONPs.

### Physicochemical properties of the prepared films

The recorded values of the thickness, oxygen transmission rate, water vapor permeability, transparency, and solubility of the prepared films with the two concentrations of ZnONPs as well as film prepared without ZnONPs were presented in Table 1. The obtained data showed that the thicknesses of the CMC-loaded NPs films were higher than that for the CMC film without NPs (Control). The increase in the ZnONPs content from 0% to 0.5% and 1.0% in the CMC film resulted in an increase in the recorded values of thickness from 0.214 mm to 0.231 mm and 0.275 mm, respectively. This increase in the recorded values of thickness could be attributed to the increased solid content and structure of the formed film between ZnONPs and CMC. As well, the addition of ZnONPs to the CMC films promoted lower oxygen transmission rates in comparison with control. The oxygen transmission rate values decreased to 2.3 × 10^–^^2^ and 1.8 × 10^–^^2^, respectively, compared with 24.7 × 10^–^^2^ for the CMC film without ZnONPs (control). Values of the water vapor permeability of the prepared films (Table 1) decreased from 0.475 g m^−^^2^ (for CMC film without NPs, control) to 0.093 g m^−^^2^ (for CMC + 0.5%ZnONPs) and 0.026 g m^−^^2^ (for CMC + 1.0%ZnONPs).

**Table 1:** Physicochemical properties of the prepared films

Film samples	Thickness (mm)	Oxygen transfer rate	Water vapors permeability (g m^−2^)	Solubility (%)	Transparency (%)
CMC (Control)	0.214 ± 0.025^b^	24.7 × 10^2^ ± 0.17^a^	0.475 ± 0.03^a^	78.31 ± 0.05^a^	84.5 ± 0.8^a^
CMC + 0.5%ZnONPs	0.231 ± 0.027^ab^	2.3×10^−2^ ± 0.02^b^	0.093 ± 0.02^b^	66.04 ± 0.01^b^	1.4 ± 0.04^b^
CMC + 1.0%ZnONPs	0.275 ± 0.001^a^	1.8×10^−2^ ± 0.09^c^	0.026 ± 0.01^c^	59.72 ± 0.01^c^	0.7 ± 0.01^c^

Calculated mean is for triplicate measurements from three independent experiments ± SD

^a–c^Means with different superscripts in the same column are considered statistically different (LSD test, *P* ≤ 0.05)

Results of testing the water solubility of the prepared films (Table 1) showed that CMC films loaded with NPs had lower values of solubility than the CMC film without NPs (control). The solubility values decreased from 78.31% (control) to 66.04% (for CMC + 0.5%ZnONPs film) and 59.72% (for CMC + 1.0%ZnONPs film).

Data of testing transparency of the prepared films (Table 1) revealed that CMC film without NPs was clear and transparent. The addition of ZnONPs influenced the appearance of the prepared films in both transparency and color and transparency. Color the two CMC loaded ZnONPs films tended to be white compared to the colorless control CMC film. Transparency was also decreased from 84.5% (for CMC control) to 1.4% and 0.7% after addition 0.5 and 1.0% ZnONPs, respectively.

### Mechanical properties of the prepared films

The recorded values of the tensile strength, elongation at break, and Young’s modulus for the of the prepared films with the two concentrations of ZnONPs as well as film prepared without NPs are shown in Table 2. The obtained data showed that there was an increase in tensile strength from 183.2 MPa (for CMC film without NPs, control) to 203.34 MPa and 235.94 MPa (for CMC + 0.5%ZnONPs and CMC + 1.0%ZnONPs, respectively). The tensile strength reached maximum value when the ZnONPs were added at a concentration of 1.0% of the CMC film. The obtained data (Table 1) also indicated that a similar change in the elongation was observed. The elongation values were increased from 13.0% (control) to 62.5% and 83.7% with ZnONPs 0.5% and 1.0%, respectively. The obtained data further indicated that the recorded value of the Young’s modulus was dramatically enhanced from 325.344 (for CMC film without NPs) to 1410.0 and 1814.96 MPa when ZnONPs was added at 0.5% and 1%, respectively.

**Table 2:** Mechanical properties of the prepared films

Film samples	Tensile strength (MPa)	Elongation (%)	Young's modulus (MPa)
CMC (Control)	183.30 ± 0.11^c^	13.00 ± 0.21^c^	325.34 ± 11.21^c^
CMC + 0.5%ZnONPs	203.34 ± 0.32^b^	62.50 ± 0.55^b^	1410.00 ± 10.34^b^
CMC + 1.0%ZnONPs	235.94 ± 0.43^a^	83.70 ± 0.37^a^	1814.96 ± 12.54^a^

Calculated mean is for triplicate measurements from three independent experiments ± SD

^a–c^Means with different superscripts in the same column  are considered statistically different (LSD test, *P* ≤ 0.05)

### Antioxidant activity of the prepared films

**Fig. 6:**
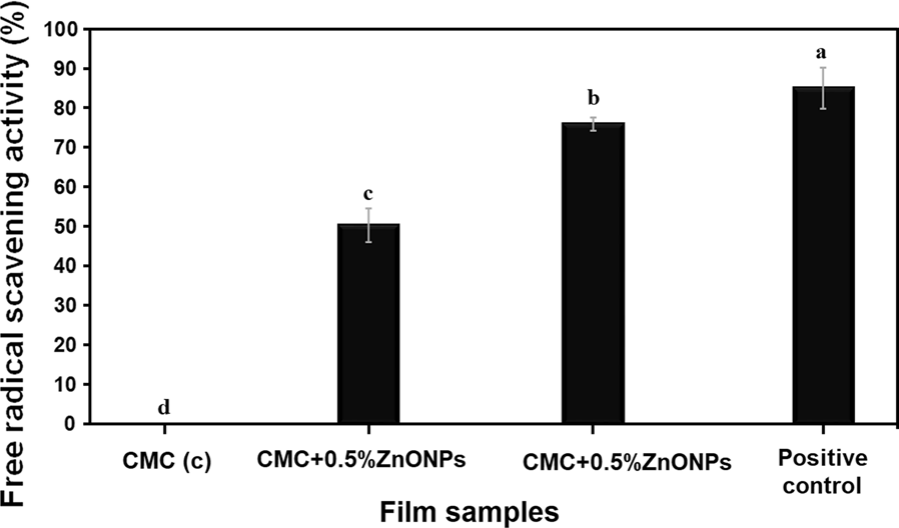
DPPH free radical scavenging activity of the prepared films. Ascorbic acid was used as positive control. Calculated mean is for triplicate measurements from three independent experiments ± SD, ^a−d^ means with different superscripts are considered statistically different (LSD test, *P* ≤ 0.05)

Figure 6 shows the recorded values obtained from testing the antioxidant behavior of the prepared films with the two concentrations of ZnONPs as well as film prepared without NPs. The obtained data indicated the significantly improved antioxidant potential of the CMC loaded ZnONPs films in comparison with CMC film without NPs. Surprisingly, the CMC loaded ZnONPs films had promising antioxidant behavior when compared to the powerful antioxidant ascorbic acid. Meanwhile, the prepared CMC film without ZnONPs showed no antioxidant activity.

### Antimicrobial activity of the prepared films

**Fig. 7:**
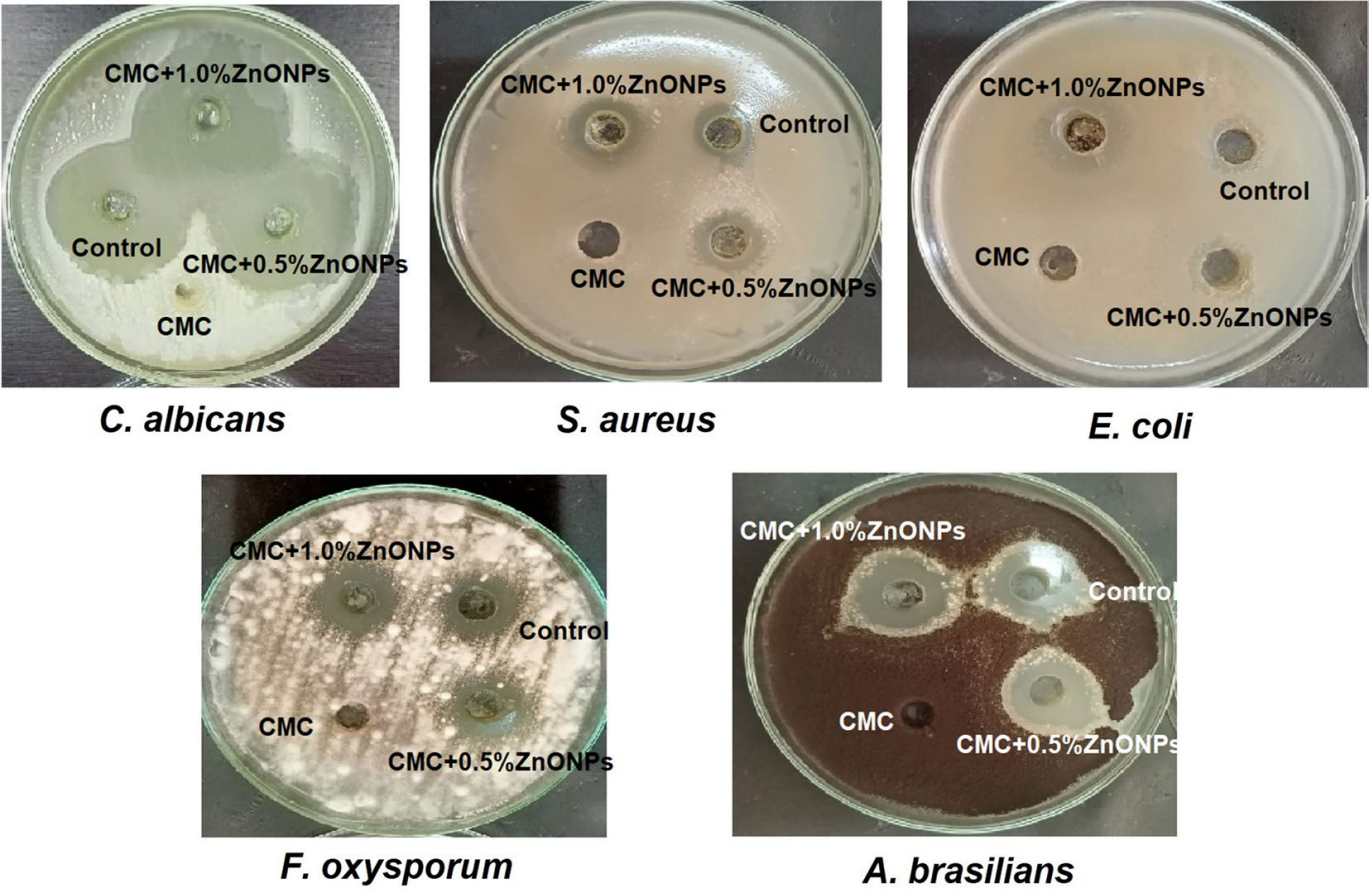
Photograph of the antibacterial and antifungal activities of the prepared films against different pathogenic bacterial strains, unicellular fungi, and plant pathogenic fungi

The antimicrobial potential of the prepared films with the two concentrations of ZnONPs as well as film prepared without NPs was evaluated against different pathogenic bacteria, unicellular fungi, and plant pathogenic fungi. Figure 7 and Table 3 clearly show that addition of ZnONPs to the CMC dramatically intensified both their antibacterial and antifungal activities, where the growth of all the tested bacteria and fungi was inhibited. Meanwhile, the CMC film without ZnONPs had no antimicrobial activity. Generally, the exact mechanism of the antimicrobial potential of ZnONPs is still unknown.

**Table 3:** Antimicrobial activity of the prepared films against different human and plant pathogenic bacterial and fungi species

Test pathogen	Diameter of inhibition zone (mm)			
	CMC	CMC + 0.5%ZnONPs	CMC + 1.0%ZnONPs	Positive controls*
*E.* *coli*	0.00^d^	17.67 ± 1.15^c^	23.00 ± 1.73^b^	28.67 ± 2.31^a^
*S.* *aureus*	0.00^d^	18.67 ± 2.31^c^	24.00 ± 1.00^b^	30.33 ± 1.15^a^
*F.* *oxysporum*	0.00^c^	22.33 ± 1.54^b^	27.67 ± 0.58^a^	28.67 ± 2.31^a^
*A.* *brasiliensis*	0.00^c^	24.67 ± 0.58^b^	28.00 ± 2.00^a^	30.00 ± 1.00^a^
*C.* *albicans*	0.00^d^	38.33 ± 2.082^c^	43.33 ± 1.15^b^	48.67 ± 2.08^a^

*Azithromycin and ketoconazole were used as positive controls at a concentration of 100 μg mL^−1^

Calculated mean is for triplicate measurements from three independent experiments ± SD

^a–d^Means with different superscripts in the same row  are considered statistically different (LSD test, *P* ≤ 0.05)

## Discussion

In this study, XRD analysis of the prepared ZnONPs confirmed the hexagonal type crystal structure with space group P36mc which is in agreement with the Joint Committee on Powder Diffraction Standards (JCPDS) card No. 361451 (Mousa et al. 2021; Abdelhakim et al. 2020; Das et al. 2013). Moreover, the synthesized NPs are pure and of a single phase where no peaks were found in the obtained pattern corresponding to impurities. Scherrer equation were used to calculate the crystallite size of ZnONPs where it was 15.61 nm. TEM analysis indicated that all the prepared particles were typically spherical in shape. Dynamic light scattering analysis proved that distribution of the prepared ZnONPs was in the range from 10 to 23 nm with a recorded polydispersity index value of 0.337 and a zeta potential value of − 25.08 mV. In general, all the previously-mentioned data obtained in this study were in a good agreement with the previous report concerning the same fungal strain (Abdelhakim et al. 2020).

Results of studying the influence of exposure of spores of the fungus to gamma rays at several doses on the obtained yield of ZnONPs demonstrated that spore suspension irradiated at 500 Gy intensified the yield of ZnONPs with a maximum yield expressed by the 0.952 OD mL^−^^1^ culture filtrate and the 81.47 mg ZnONPs 100 mL^−^^1^ culture filtrate; representing approximately a fourfold increase. In partial accordance with these results, a previous study used the same methodology of gamma irradiation but at a different dose (1000 Gy) increase the yield of ZnONPs by the fungus *Aspergillus* *terreus* (El-Sayed et al. 2022b). In previous studies, irradiating spores of the fungus *Monascus* *purpureus* by gamma rays at 1000 Gy significantly intensified the yields of both cobalt-ferrite NPs (El-Sayed et al. 2020b) and selenium NPs (El-Sayed et al. 2020a). In generally, the observed increase in the prepared ZnONPs yield could be mainly attributed to the gamma ray’s exposure of the fungal cells prior to the preparation of the cell-free filtrate used in the preparation of NPs. Exposure of the fungus cells to gamma rays might induced mutations (El-Sayed et al. 2022a; Hazaa et al. 2022) in these cells resulting in the over-production of all the active metabolites and enzymes responsible (El-Sayed et al. 2022c; El-Sayed and Zaki 2022) for the bio-reduction of zinc sulfate salt and intensifying the formation of high concentrations of ZnONPs. Previous reports showed that the irradiation by gamma rays at some specific doses was successfully used for enhancing the production of various fungal metabolites (El-Sayed 2021; Zaki and El-Sayed 2021; El-Sayed et al. 2019a, b, c; El-Sayed et al. 2020c, d, e, f).

In the current study, the synthesized ZnONPs were exploited to develop active CMC films by casting method. Values of the thickness, oxygen transmission rate, water vapor permeability, transparency, and solubility of the prepared films with the two concentrations of ZnONPs as well as film prepared without ZnONPs were evaluated. The obtained data showed that the thicknesses of the CMC-loaded NPs films were higher than that for the CMC film without NPs (Control). The increase in the ZnONPs content from 0% to 0.5% and 1.0% in the CMC film resulted in an increase in the recorded values of thickness. This increase in the recorded values of thickness could be attributed to the increased solid content and structure of the formed film between ZnONPs and CMC (Jebel and Almasi 2016). As such, the addition of ZnONPs to the CMC films promoted lower oxygen transmission rates in comparison with control. This reduction in the oxygen transmission rates is mainly due to the distribution of NPs in the film matrix, thereby reducing the diffusion of oxygen (Ngo et al., 2018). Similarly, Basumatary et al. (2020) concluded that reinforcement of biopolymers with nanomaterials such as ZnONPs and AgNPs improved physicochemical, mechanical, barrier properties. The authors further reported that ZnONPs 2% loaded PLA film resulted in improvement in tensile strength and oxygen barrier properties by 29% and 25%, respectively. Our results indicated the capability of the prepared CMC loaded ZnONPs films to inhibit oxidation that will promote their role in the food preservation. Values of the water vapor permeability of the prepared films decreased from 0.475 g m^−^^2^ (for CMC film without NPs, control) to 0.093 g m^−^^2^ (for CMC + 0.5%ZnONPs) and 0.026 g m^−^^2^ (for CMC + 1.0%ZnONPs). In accordance with our results, Hajizadeh (2020) Incorporating MO_2_@Ag NPs into starch matrix decreased solubility in water and water vapor permeability of the obtained films. This decrease in the permeability of the prepared films could be attributed to the formation of the hydrogen bonds between matrix of the CMC polymer and oxygen atoms in the ZnONPs. The formation of new hydrogen bonds could increase the adhesion of the CMC film thereby reducing the water diffusion in the CMC matrix (Almasi et al. 2010). Furthermore, the even distribution of ZnONPs with extremely low particle size as well as filling the microvoids of the CMC matrix reduced the length of a free path for the water uptake. In accordance with previous studies, the same observations were reported for the effect of ZnONPs on the water vapor permeability of biopolymer laded films (Almasi et al. 2010; Ngo et al. 2018; Basumatary et al. 2020; Anugrah et al. 2020). Results of testing the water solubility of the prepared films showed that CMC films loaded with NPs had lower values of solubility than the CMC film without NPs (control). The addition of the ZnONPs decreased the number of hydroxyl groups and charged groups and increased the length of the polymer due to the formation of links between the CMC matrix and ZnONPs thereby reducing the solubility of the films (Almasi et al. 2010). Generally, CMC has good reactivity and solubility and reactivity due to the reactive hydroxyl and carboxyl functional groups (Anugrah et al. 2020). Water solubility of packaging films is beneficial for some food products such as products that are heated before consumption. In addition, the water solubility of a packaging film facilitates its biodegradability (Shah et al. 2008). Conversely, films with high solubility are not suitable for packaging food products with high humidity, so it is preferable to reduce their solubility by adding NPs. Transparency is an important index in terms of general appearance and consumer acceptance. The addition of ZnONPs influenced the appearance of the prepared films in both transparency and color and transparency. Similarly, Nafchi et al. (2012) noticed that addition of BEO and ZnONPs to the FPI/FSG films lowered their transparency. Also, Yoo and Krochta (2011) reported that the gelatin/ZnONPs films had a lower transparency with a slight greenish-yellow tint, therefore, lowering the total color difference value following ZnONPs and gelatin amalgamation.

In the present study, mechanical properties of the tensile strength, elongation at break, and Young’s modulus for the of the prepared films with the two concentrations of ZnONPs as well as film prepared without NPs were also studied. The obtained data showed that tensile strength reached maximum value when the ZnONPs were added at a concentration of 1.0% of the CMC film. Changes in both tensile strength and elongation could be attributed to the conjugation between atoms of the ZnONPs and the CMC groups (Almasi et al. 2010). This conjugation resulted in strong interactions among the CMC matrix and the added NPs which increased crystallinity of the film's structure (Azizi et al. 2014). The obtained data further indicated that the recorded value of the Young’s modulus was dramatically enhanced. Generally, Young’s modulus as useful measures of the film brittleness and stiffness, extensibility, and mechanical strength are typically required for the maintenance of the barrier properties and physical integrity of packaging films to mitigate external forces during processing and preservation food products (Zahedim et al. 2018). Accordingly, improvement of the mechanical resistance of the prepared films in this study against external stresses might promote the possibility of full replacement with a synthetic polymer.

Results of testing the antioxidant behavior of the prepared films with the two concentrations of ZnONPs as well as film prepared without NPs indicated the improved antioxidant potential of the CMC loaded ZnONPs films in comparison with CMC film without NPs. Surprisingly, the CMC loaded ZnONPs films had promising antioxidant behavior, although the prepared CMC film without ZnONPs showed no antioxidant activity. In the literature, several studies reported the promising free radical scavenging potential of various metal NPs (Mousa et al. 2021; Abdelhakim et al. 2020; Kovacic and Somanathan 2013; and references therein). Generally, the antioxidant potential of nanomaterials was accredited to the high surface-to-volume ratio. As such, the inhibition and neutralization by NPs of free radicals generated by the DPPH (Mousa et al., 2021).

Here, data regarding the antimicrobial potential of the prepared films as well as film prepared without NPs against different pathogenic bacteria, unicellular fungi, and plant pathogenic fungi clearly revealed that addition of ZnONPs to the CMC dramatically intensified both their antibacterial and antifungal activities. In agreement with our results, Peighambardoust et al. (2019) found that use of Ag/ZnO/CuO NPs in the formulation of starch-based nanocomposite films exhibit synergistic effect in enhancing the antimicrobial effect against *S.* *aureus* and *E.* *coli*. Also, Hajizadeh et al. (2020) Incorporating MO_2_@Ag NPs into starch matrix significantly inhibited the growth of *Escherichia* *coli* and *Staphylococcus* *aureus*. The authors further concluded that the most antibacterial effect was obtained for the films containing higher weight concentrations of Ag-loaded SiO_2_-NPs. Generally, the exact mechanism of the antimicrobial potential of ZnONPs is still unknown. In one hand, some reports attributed this activity to the inhibitory effect to cellular growth resulted from the increasing intracellular production of reactive oxygen species (Abdelhakim et al. 2020; Mousa et al. 2021). On the other hand, some studies attributed this antimicrobial effect to the quick interaction of NPs with the cell membrane or cell wall causing leakage of cellular components such as genetic materials, proteins, and minerals resulting in cell death (El-Sayed et al. 2020a, b; Hussien et al. 2022).

In summary, the yield of Zinc oxide nanoparticles synthesized using *Alternaria* *tenuissima* was intensified by gamma irradiation to approximately four-fold which could open up the way for the manufacture at an industrial scale using a cost-effective and green technology. Furthermore, active CMC films were successfully developed using the synthesized NPs. The developed CMC films showed promising activities in comparison with CMC films without NPs. These findings suggest the effective use of the CMC-loaded ZnONPs films as protective edible coatings to improve the quality and shelf life of food products thereby promoting the feasibility of full replacement with the synthetic polymers.

## Data Availability

The authors confirm that the data supporting the findings of this study are available within the article.

## References

[CR1] Abdelhakim HK, El-Sayed ER, Rashidi FB (2020) Biosynthesis of zinc oxide nanoparticles with antimicrobial, anticancer, antioxidant and photocatalytic activities by the endophytic *Alternaria**tenuissima*. J Appl Microbiol 128:1634–1646. 10.1111/jam.14581 31954094 10.1111/jam.14581

[CR2] Almasi H, Ghanbarzadeh B, Entezami AA (2010) Physicochemical properties of starch-CMC-nanoclay biodegradable films. Int J Biol Macromol 46:1–5. 10.1016/j.ijbiomac.2009.10.001 19828115 10.1016/j.ijbiomac.2009.10.001

[CR3] Anugrah DSB, Alexander H, Pramitasari R, Hudiyanti D, Sagita CB (2020) A review of polysaccharide-zinc oxide nanocomposites as safe coating for fruits preservation. Coatings 10:988. 10.3390/coatings10100988

[CR4] ASTM (1990) Standard practice for conditioning plastics and electrical insulating materials for testing: D618-61 (reapproved 1990). In: Annual book of American Standard Testing Methods, Vol. 8.01. (1995) Philadelphia.

[CR5] Azizi S, Ahmad MB, Hussein MZ, Ibrahim NA, Namvar F (2014) Preparation and properties of poly (vinyl alcohol)/chitosan blend bionanocomposites reinforced with cellulose nanocrystals/ZnO-Ag multifunctional nanosized filler. Int J Nanomed 9:1909–1917. 10.2147/IJN.S60274 10.2147/IJN.S60274 PMC4003268 24790433

[CR6] Basumatary IB, Mukherjee A, Katiyar V, Kumar S (2020) Biopolymer-based nanocomposite films and coatings: recent advances in shelf-life improvement of fruits and vegetables. Crit Rev Food Sci Nutr. 10.1080/10408398.2020.1848789 33249872 10.1080/10408398.2020.1848789

[CR7] Bisht G, Rayamajhi S (2016) ZnO nanoparticles: a promising anticancer agent. Nanobiomedicine 3:9. 10.5772/63437 29942384 10.5772/63437 PMC5998263

[CR8] Das D, Nath BC, Phukon P, Dolui SK (2013) Synthesis of ZnO nanoparticles and evaluation of antioxidant and cytotoxic activity. Colloids Surf B 111:556–560. 10.1016/j.colsurfb.2013.06.041 10.1016/j.colsurfb.2013.06.041 23891844

[CR9] Dehghania S, Peighambardousta SH, Peighambardoust SJ, Hosseini VL, Joe Regenstein JM (2019) Improved mechanical and antibacterial properties of active LDPE films prepared with combination of Ag, ZnO and CuO nanoparticles. Food Packag Shelf Life 22:100391. 10.1016/j.fpsl.2019.100391

[CR10] Ebrahimi Y, Peighambardoust SJ, Peighambardoust SH, Karkaj SZ (2019) Development of antibacterial carboxymethyl cellulose-based nanobiocomposite films containing various metallic nanoparticles for food packaging applications. J Food Sci 84:2537–2548. 10.1111/1750-3841.14744 31433502 10.1111/1750-3841.14744

[CR11] El-Sayed ER (2021) Discovery of the anticancer drug vinblastine from the endophytic *Alternaria**alternata* and yield improvement by gamma irradiation mutagenesis. J Appl Microbiol 131:2886–2898. 10.1111/jam.15169 34062037 10.1111/jam.15169

[CR12] El-Sayed ER, Zaki AG (2022) Unlocking the biosynthetic potential of *Penicillium**roqueforti* for hyperproduction of the immunosuppressant mycophenolic acid: gamma radiation mutagenesis and response surface optimization of fermentation medium. Biotechnol Appl Biochem. 10.1002/bab.2353 35481612 10.1002/bab.2353

[CR13] El-Sayed ER, Ahmed AS, Ismaiel AA (2019a) Agro-industrial byproducts for production of the immunosuppressant mycophenolic acid by *Penicillium**roqueforti* under solid-state fermentation: enhanced production by ultraviolet and gamma irradiation. Biocatal Agric Biotechnol 18:101015. 10.1016/j.bcab.2019.01.053

[CR14] El-Sayed ER, Ismaiel AA, Ahmed AS, Hassan IA, Karam El-Din AA (2019b) Bioprocess optimization using response surface methodology for production of the anticancer drug paclitaxel by *Aspergillus**fumigatus* and *Alternaria**tenuissima*: enhanced production by ultraviolet and gamma irradiation. Biocatal Agric Biotechnol 18:100966. 10.1016/j.bcab.2019.01.034

[CR15] El-Sayed ER, Ahmed AS, Hassan IA, Ismaiel AA, Karam El-Din AA (2019c) Strain improvement and immobilization technique for enhanced production of the anticancer drug paclitaxel by *Aspergillus**fumigatus* and *Alternaria**tenuissima*. Appl Microbiol Biotechnol 103:8923–8935. 10.1007/s00253-019-10129-1 31520132 10.1007/s00253-019-10129-1

[CR16] El-Sayed ER, Abdelhakim HK, Ahmed AS (2020a) Solid–state fermentation for enhanced production of selenium nanoparticles by gamma-irradiated *Monascus**purpureus* and their biological evaluation and photocatalytic activities. Bioproc Biosyst Eng 43:797–809. 10.1007/s00449-019-02275-7 10.1007/s00449-019-02275-7 31898764

[CR17] El-Sayed ER, Abdelhakim HK, Zakaria Z (2020b) Extracellular biosynthesis of cobalt ferrite nanoparticles by *Monascus**purpureus* and their antioxidant, anticancer and antimicrobial activities: yield enhancement by gamma irradiation. Mater Sci Eng C 107:110318. 10.1016/j.msec.2019.110318 10.1016/j.msec.2019.110318 31761250

[CR18] El-Sayed ER, Ahmed AS, Abdelhakim HK (2020c) A novel source of the cardiac glycoside digoxin from the endophytic fungus *Epicoccum**nigrum*: Isolation, characterization, production enhancement by gamma irradiation mutagenesis and anticancer activity evaluation. J Appl Microbiol 128:747–762. 10.1011/JAM.14510 31710165 10.1111/jam.14510

[CR19] El-Sayed ER, Zaki AG, Ahmed AS, Ismaiel AA (2020d) Production of the anticancer drug taxol by the endophytic fungus *Epicoccum**nigrum* TXB502: enhanced production by gamma irradiation mutagenesis and immobilization technique. Appl Microbiol Biotechnol 104:6991–7003. 10.1007/s00253020-10712-x 32617617 10.1007/s00253-020-10712-x

[CR20] El-Sayed ER, Ahmed AS, Al-Hagar OEA (2020e) Agro-industrial wastes for production of paclitaxel by irradiated *Aspergillus**fumigatus* under solid-state fermentation. J Appl Microbiol 128:1427–1439. 10.1111/jam.14574 31912646 10.1111/jam.14574

[CR21] El-Sayed ER, Ahmed AS, Hassan IA, Ismaiel AA, Karam El-Din AA (2020f) Semi-continuous production of the anticancer drug taxol by *Aspergillus**fumigatus* and *Alternaria**tenuissima* immobilized in calcium alginate beads. Bioprocess Biosyst Eng 43:997–1008. 10.1007/s00449-020-02295-8 31997009 10.1007/s00449-020-02295-8

[CR22] El-Sayed ER, Hazaa MA, Shebl MA, Amer MM, Mahmoud SR, Khattab AA (2022a) Bioprospecting endophytic fungi for bioactive metabolites and use of irradiation to improve their bioactivities. AMB Expr 12:46. 10.1186/s13568-022-01386-x 10.1186/s13568-022-01386-x PMC9018947 35438322

[CR23] El-Sayed ER, Mousa SA, Abdou DAM, Abo El-Seoud MA, Elmehlawy AA, Mohamed SS (2022b) Exploiting the exceptional biosynthetic potency of the endophytic *Aspergillus**terreus* in enhancing production of CO_3_O_4_, CuO, Fe_3_O_4_, NiO, and ZnO nanoparticles using bioprocess optimization and gamma irradiation. Saudi J Biol Sci 29:2463–2474. 10.1016/j.sjbs.2021.12.019 35531225 10.1016/j.sjbs.2021.12.019 PMC9072909

[CR24] El-Sayed ER, Gach J, Olejniczak T, Boratyński F (2022c) A new endophyte *Monascus**ruber* SRZ112 as an efficient production platform of natural pigments using agro-industrial wastes. Sci Rep 12:12611. 10.1038/s41598-022-16269-1 35871189 10.1038/s41598-022-16269-1 PMC9308793

[CR25] Fasihnia SH, Peighambardoust SH, Peighambardoust SJ, Oromiehie A (2018) Development of novel active polypropylene-based packaging films containing different concentrations of sorbic acid. Food Packag Shelf Life 18:87–94. 10.1016/j.fpsl.2018.10.001

[CR26] Hajizadeh H, Peighambardoust SJ, Peighambardoust SH, Peressini D (2020) Physical, mechanical, and antibacterial characteristics of bio-nanocomposite films loaded with Ag-modified SiO_2_ and TiO_2_ nanoparticles. J Food Sci 85:1193–1202. 10.1111/1750-3841.15079 32144762 10.1111/1750-3841.15079

[CR27] Han Y, Wang L (2017) Sodium alginate/carboxymethyl cellulose films containing pyrogallic acid: physical and antibacterial properties. J Sci Food Agric 97:1295–1301. 10.1002/jsfa.7863 27328858 10.1002/jsfa.7863

[CR28] Hazaa AA, Shebl MM, EEl-Sayed ER, Mahmoud SR, Khattab AA, Amer MM (2022) Bioprospecting endophytic fungi for antifeedants and larvicides and their enhancement by gamma irradiation. AMB Express 10.1186/s13568-022-01461-3 PMC9481848 36114376

[CR29] Hernandez-Mun P, Villalobos R, Chiralt A (2004) Effect of cross-linking using aldehydes on properties of glutenin-rich films. Food Hydrocoll 18:403. 10.1016/S0268-005X(03)00128-0

[CR30] Hosseini MR, Sarviab MN (2015) Recent achievements in the microbial synthesis of semiconductor metal sulfide nanoparticles. Mater Sci Semicond Process 40:293–301. 10.1016/j.mssp.2015.06.003

[CR31] Hussein HG, El-Sayed ER, Younis NA, Hamdy AA, Easa SM (2022) Harnessing endophytic fungi for biosynthesis of selenium nanoparticles and exploring their bioactivities. AMB Expr 12:68. 10.1186/s13568-022-01408-8 10.1186/s13568-022-01408-8 PMC9177918 35674975

[CR32] Jebel SF, Almasi H (2016) Morphological, physical, antimicrobial and release properties of ZnO nanoparticles-loaded bacterial cellulose films. Carbohydr Polym 149:8–19. 10.1016/j.carbpol.2016.04.089 27261725 10.1016/j.carbpol.2016.04.089

[CR33] Khodaeimehr R, Peighambardoust SJ, Peighambardoust SH (2018) Preparation and characterization of corn starch/clay nanocomposite films: effect of clay content and surface modification. Starch 70:1700251. 10.1002/star.201700251

[CR34] Kovacic P, Somanathan R (2013) Nanoparticles: toxicity, radicals, electron transfer, and antioxidants. In Armstrong, D., Bharali, D. (Eds) Oxidative Stress and Nanotechnology. Methods in Molecular Biology (Methods and Protocols), vol 1028. Humana Press, Totowa, NJ. 10.1007/978-1-62703-475-3_2 10.1007/978-1-62703-475-3_2 23740111

[CR35] La DD, Nguyen-Tri P, Le KH, Nguyen PTM, Nguyen MD, Vo ATK, Nguyen MTH, Chang SW, Tran LD, Chung WJ, Nguyen DD (2021) Effects of antibacterial ZnO nanoparticles on the performance of a chitosan/gum arabic edible coating for post-harvest banana preservation. Prog Org Coat 151:106057. 10.1016/j.porgcoat.2020.106057

[CR36] Mousa SA, El-Sayed ER, Mohamed SS, Abo El-Seoud MA, Elmehlawy AA, Abdou DAM (2021) Novel mycosynthesis of Co_3_O_4_, CuO, Fe_3_O_4_, NiO, and ZnO nanoparticles by the endophytic *Aspergillus**terreus* and evaluation of their antioxidant and antimicrobial activities. Appl Microbiol Biotechnol 105:741–753. 10.1007/s00253-020-11046-4 33394153 10.1007/s00253-020-11046-4

[CR37] Nafchi AM, Alias AK, Mahmud S, Robal M (2012) Antimicrobial, rheological, and physicochemical properties of sago starch films filled with nanorod-rich zinc oxide. J Food Eng 113:511–519. 10.1016/j.jfoodeng.2012.07.017

[CR38] Ngo TMP, Dang TMO, Tran TX, Rachtanapun B (2018) Effects of zinc oxide nanoparticles on the properties of pectin/alginate edible films. Int J Polym Sci 2018:5645797. 10.1155/2018/5645797

[CR39] Oun AA, Rhim JW (2017) Preparation of multifunctional chitin nanowhiskers/ZnO-Ag NPs and their effect on the properties of carboxymethyl cellulose-based nanocomposite film. Carbohydr Polym 169:467–479. 10.1016/j.carbpol.2017.04.042 28504170 10.1016/j.carbpol.2017.04.042

[CR40] Peighambardoust SJ, Pourabbas B (2007) Preparation and characterization of nylon-6/PPy/MMT composite of nanocomposite. J Appl Polym Sci 106:697–705. 10.1002/app.26709

[CR41] Peighambardoust SJ, Peighambardoust SH, Pournasir N, Pakdel PM (2019) Properties of active starch-based films incorporating a combination of Ag, ZnO and CuO nanoparticles for potential use in food packaging applications. Food Packag Shelf Life 22:100420. 10.1016/j.fpsl.2019.100420

[CR42] Sahraeea S, Milanib JM, Regensteinc GM, Kafild HS (2019) Protection of foods against oxidative deterioration using edible films and coatings: a review. Food Biosci 32:100451. 10.1016/j.fbio.2019.100451

[CR43] Shah A, Hasan AF, Hameed A, Ahmed S (2008) Biological degradation of plastics: a comprehensive review. Biotechnol Adv 26:246–265. 10.1016/j.biotechadv.2007.12.005 18337047 10.1016/j.biotechadv.2007.12.005

[CR44] Shahid S, Fatima U, Sajjad R, Khan SA (2019) Bioinspired nanotheranostic agent: zinc oxide; green synthesis and biomedical potential. Digest J Nanomater Biostruct 14:1023–1031

[CR46] Tien CL, Letendre M, Ispas-Szabo P, Mateescu MA, Delmas-Patterson G, Yu HL, Lacroix M (2000) Development of biodegradable films from whey proteins by cross-linking and entrapment in cellulose. J Agric Food Chem 48:5566. 10.1021/jf0002241 11087520 10.1021/jf0002241

[CR47] Wajiha QNA, Afridi R (2018) Comparative analysis of egg adapted vaccines and salinomycin against coccidiosis in chicks. Microb Pathog 123:454–460. 10.1016/J.MICPATH.2018.08.005 30086345 10.1016/j.micpath.2018.08.005

[CR48] Yoo S, Krochta JM (2011) Whey protein-polysaccharide blended edible film formation and barrier, tensile, thermal and transparency properties. J Sci Food Agric 91:2628–2636. 10.1002/jsfa.4502 21717463 10.1002/jsfa.4502

[CR49] Yusof HM, Mohamad R, Zaidan UH, Abdul Rahman N (2019) Microbial synthesis of zinc oxide nanoparticles and their potential application as an antimicrobial agent and a feed supplement in animal industry: a review. J Anim Sci Biotechnol 10:57. 10.1186/s40104-019-0368-z 31321032 10.1186/s40104-019-0368-z PMC6615095

[CR50] Zahedim Y, Fathi-Achachlouei B, Yousefi AR (2018) Physical and mechanical properties of hybrid montmorillonite/zinc oxide reinforced carboxymethyl cellulose nanocomposites. Int J Biol Macromol 108:863–873. 10.1016/j.ijbiomac.2017.10.185 29102792 10.1016/j.ijbiomac.2017.10.185

[CR51] Zaki AG, El-Sayed ER (2021) New and potent production platform of the acetylcholinesterase inhibitor Huperzine A by gamma-irradiated *Alternaria**brassicae* under solid-state fermentation. Appl Microbiol Biotechnol 105:8869–8880. 10.1007/s00253-021-11678-0 34748037 10.1007/s00253-021-11678-0

[CR52] Zambrano-Zaragoza ML, González-Reza R, Mendoza-Muñoz N, Miranda-Linares V, Bernal-Couoh TF, Mendoza-Elvira S, Quintanar-Guerrer D (2018) Nanosystems in edible coatings: a novel strategy for food preservation. Int J Mol Sci 19:705. 10.3390/ijms19030705 29494548 10.3390/ijms19030705 PMC5877566

