# Correction: Improving carboxymethyl cellulose edible coating using ZnO nanoparticles from irradiated *Alternaria tenuissima*

**DOI:** 10.1186/s13568-024-01779-0

**Published:** 2024-11-14

**Authors:** Mervat M. Anwar, Sanaa S. H. Aly, Essam H. Nasr, El‑Sayed R. El‑Sayed

**Affiliations:** 1 Plant Research Department, Nuclear Research Center, Egyptian Atomic Energy Authority, Cairo, Egypt; 2 Food Engineering and Packaging Department, Agriculture Research Centre, Food Technology Research Institute, Giza, Egypt

**Correction to: AMB Express (2022) 12:116** 10.1186/s13568-022-01459-x

Following publication of the original article (Anwar et al. 2022), the author “Sanaa S. H. Aly” noticed that the organization name was missing for the second affiliation. The organization name “Agriculture Research Centre” has updated with this erratum.

**Incorrect affiliation:**

Food Engineering and Packing Department, Food Technology Research Institute, Giza, Egypt.

**Corrected affiliation**

Food Engineering and Packing Department, Agriculture Research Centre, Food Technology Research Institute, Giza, Egypt.

The original article has been corrected.

## References

[CR1] Anwar MM, Aly SSH, Nasr EH et al (2022) Improving carboxymethyl cellulose edible coating using ZnO nanoparticles from irradiated *Alternaria tenuissima* AMB Expr 12:116 10.1186/s13568-022-01459-x PMC9452608 36070053

